# Sexual Neurasthenia

**Published:** 1891-09

**Authors:** 


Sexual Neurasthenia (Nervous Exhaustion), its Hygiene, Causes, Symptoms, and Treatment, with a chapter on Diet for the Nervous. By George M. Beard, A. M., M. D., formerly Lecturer on Nervous Diseases in the University of the City of New York; Fellow of the New York Academy of Medicine; author of "Our Home Physician," "Hay Fever;" one of the Authors of "Medical and Surgical Electricity," etc. (Posthumous Manuscript). Edited by A. D. Rockwell, A. M., M. D.—Professor of Electro-Therapeutics N. Y. Post-Graduate Medical School and Hospital; Fellow of the New York Academy of Medicine, and one of the Authors of *Medical and Surgical Electricity*. In one volume, Crown 8vo, nearly 300 pages. $2.75. E. B. Treat, Publisher, 5 Cooper Union, N. Y.

The philosophy of this work is based on the theory that there is a special and very important and very frequent clinical variety of neurasthenia (nervous exhaustion) to which the term sexual neurasthenia (sexual exhaustion) may properly be applied.

While this variety may be, and often is involved as a cause or effect or coincident with other varieties—exhaustion of the brain, of the spine, of the stomach, and digestive system—yet in its full development it can be and should be differentiated from hysteria, simple hypochondria, insanity, and various organic diseases of the nervous system, with all of which it had until lately been confounded.

The long familiar local conditions of genital debility in the male—impotence and spermatorrhœa, prostatorrhœa, irritable prostrate—which have hitherto been almost universally described as diseases by themselves, are philosophically and clinically analyzed. These symptoms, as such, do not usually exist alone, but are associated with other local or general symptoms of sexual neurasthenia herein described.

The causes of sexual neurasthenia are not single or simple, but complex; evil habits, excesses, tobacco, alcohol, worry, and special excitements, even climate itself, are the great predisposing causes.

The subject is restricted mainly to sexual exhaustion, as it exists in the male, for the reason that the symptoms of neurasthenia, as it exists in females, are, and for a long time have been understood and recognized. Cases analagous to those in females are dismissed as hypochondriacs, just as females suffering from now clearly explained uterine and ovarian disorders were formerly dismissed as hysterics.

This view of the relation of the reproductive system to nervous diseases is in accordance with facts that are verifiable and abundant; that in men as in women, a large group of nervous symptoms, which are very common indeed, would not exist but for morbid states of the reproductive system.—[*From Dr. Beard's Introduction.*]

The causes and symptoms of forty-three cases are given, followed by a chapter on Diet for the Nervous, with Treatment and Formulas. Third Edition Enlarged.

